# The Curcumin Analog EF24 Targets NF-κB and miRNA-21, and Has Potent Anticancer Activity In Vitro and In Vivo

**DOI:** 10.1371/journal.pone.0071130

**Published:** 2013-08-07

**Authors:** Chuan He Yang, Junming Yue, Michelle Sims, Lawrence M. Pfeffer

**Affiliations:** Department of Pathology and Laboratory Medicine and the Center for Cancer Research, University of Tennessee Health Science Center, Memphis, Tennessee, United States of America; Medical College of Wisconsin, United States of America

## Abstract

EF24 is a curcumin analog that has improved anticancer activity over curcumin, but its therapeutic potential and mechanism of action is unknown, which is important to address as curcumin targets multiple signaling pathways. EF24 inhibits the NF-κB but not the JAK-STAT signaling pathway in DU145 human prostate cancer cells and B16 murine melanoma cells. EF24 induces apoptosis in these cells apparently by inhibiting miR-21 expression, and also enhances the expression of several miR-21 target genes, PTEN and PDCD4. EF24 treatment significantly suppressed the growth of DU145 prostate cancer xenografts in immunocompromised mice and resulted in tumor regression. EF24 enhanced the expression of the miR-21 target PTEN in DU145 tumor tissue, but suppressed the expression of markers of proliferating cells (cyclin D1 and Ki67). In syngeneic mice injected with B16 cells, EF24 treatment inhibited the formation of lung metastasis, prolonged animal survival, inhibited miR-21 expression and increased the expression of miR-21 target genes. Expression profiling of miRNAs regulated by EF24 *in vitro* and *in vivo* showed that the antitumor activity of EF24 reflected the enhanced expression of potential tumor suppressor miRNAs as well as the suppressed expression of oncogenic miRNAs, including miR-21. Taken together, our data suggest that EF24 is a potent anticancer agent and selectively targets NF-κB signaling and miRNA expression, indicating that EF24 has significant potential as a therapeutic agent in various cancers.

## Introduction

Among men in the United States prostate cancer is the second most common cancer, the most common malignant disease and the second leading cause of cancer-related death [1]. Melanoma is a highly aggressive form of skin cancer, which is the most common human cancer worldwide, and melanoma accounts for ∼80% of skin cancer-related deaths in the US [2]. Although a limited number of treatment options for prostate cancer and melanoma presently exist, patients often relapse with a more aggressive form of the disease for which no effective treatment presently exists. Thus, new therapeutic strategies are needed for the treatment of prostate cancer and melanoma.

MicroRNAs (miRNAs) are short noncoding RNAs that post-transcriptionally regulate the expression of multiple genes. Although miRNAs regulate many important physiological processes, the dysregulation of miRNA expression in cancer is well established [3]. In particular miR-21 is overexpressed in various human cancers and plays an important role in cancer development, progression and metastasis [4]. We previously showed that miR-21 expression regulates the sensitivity of prostate cancer cells to chemotherapeutic drugs [5]. While knockdown of miR-21 expression in DU145 prostate cancer cells markedly enhanced sensitivity to apoptosis induced by several chemotherapeutic agents, enforced miR-21 expression in the low miR-21-expressing PC3 prostate cancer cell line markedly reduced sensitivity to apoptosis.

Diet plays a major role in many cancers, and curcumin, which is a phytochemical component of the spice turmeric, has efficacy in preclinical and clinical studies as an anticancer agent [6], [7]. Curcumin inhibits the STAT3 and NF-κB signaling pathways that appear to play important roles in cancer development and progession [8]. For example, constitutive activation of the STAT3 and NF-κB signaling pathways is found in prostate cancer cell lines and clinical samples of prostate cancer [9], [10]. The low cancer killing potency of curcumin, its multiple biological effects, and its low bioavailability has led to the development of curcumin analogs with similar safety profiles but increased anticancer activity and solubility. EF24 (diphenyl difluoroketone) is one such analog, which exhibits anticancer activity in colon and gastric cancer [11]. In the present study we show that EF24 inhibits the NF-κB but not the JAK-STAT signaling pathway in prostate cancer and melanoma cells *in vitro*, induces apoptosis, and enhances the expression of miR-21 target genes, PTEN and PDCD4. *In vivo* EF24 suppressed the growth of DU145 prostate cancer xenografts and the formation of lung metastasis in mice injected with B16 melanoma cells, and reduced miR-21 expression and enhanced the expression of miR-21 target genes in tumor tissue. Expression profiling of miRNAs showed that EF24 enhanced the expression of potential tumor suppressor miRNAs, and inhibited the expression of oncogenic miRNAs, including miR-21.

## Materials and Methods

### Biological Reagents and Cell Culture

The biological activity of recombinant human IFNα (InterMune) and murine IFN (Biogen-Idec) was expressed in terms of international reference units/ml using the human and murine NIH reference standards, respectively [12]. EF24, purchased from Sigma, was dissolved in dimethyl sulfoxide at 1 mM, stored at 4°C, and diluted in cell culture medium immediately prior to use. DU145 prostate cancer and B16 melanoma cells (obtained from ATCC) were grown as monolayers in RPMI 1640 medium with 10% fetal calf serum (Hyclone) supplemented with glutamine, penicillin, and streptomycin, and subcultured every 3 days at 10–30% confluence. Stable pools of DU145 and B16 cells transduced with antagomiR-21 lentivirus resulting in a >80% knockdown of miR-21 expression have been previously described [5], [13]. The antibodies used are listed in Table 1.

**Table 1:** Antibodies used.

Antibody	Source	Manufacturer	Catalogue number
PTEN	Rabbit	Millipore	#04-409
PDCD4	Rabbit	Rockland	#600-401-965
pSTAT3 (Tyr705)	Rabbit	Cell Signaling	#9145
STAT3	Mouse	BD Transduction	#610190
IκBα	Rabbit	Santa Cruz	#sc-371
PARP	Rabbit	Cell Signaling	#9542
Ki67	Rabbit	Abcam	ab16667
Cyclin D1	Rabbit	Abcam	ab2429

### Detection of miRNA and mRNA Expression

Gene expression was determined by quantitative real time PCR (qPCR) as previously described using gene-specific primers listed in Table 2 and 3 [5]. In brief, total RNA was isolated using RNeasy Mini kit (Qiagen). For miRNA expression, total RNA (5 µg) was reverse-transcribed into first-strand cDNA and 40 ng of cDNA was used as a template for the PCR reaction with a forward primer specific to the mature miRNA sequence as previously described [5]. SYBR Green-based real-time PCR was performed on a BioRad iCycler and gene expression normalized relative to U6 or β-actin expression for miRNA or mRNA, respectively.

**Table 2:** Forward Primers Used for miRNA Expression Analysis.

miRNAs	Forward primer sequences
miR-21	5′-TAGCTTATCAGACTGATGTTGA-3′
miR-100	5′-AACCCGTAGATCCGAACTTGTG-3′
miR-126	5′-TCGTACCGTGAGTAATAATGCG-3′
miR-181a	5′-AACATTCAACGCTGTCGGTGAGT-3
miR-200a	5′-TAACACTGTCTGGTAACGATGT-3′
miR-26a	5′-TTCAAGTAATCCAGGATAGGCT-3′
miR-24	5′-TGGCTCAGTTCAGCAGGAACAG-3′
miR-30b	5′-TGTAAACATCCTACACTCAGCT-3′
miR-29a	5′-TAGCACCATCTGAAATCGGTTA-3′
miR-10a	5′-TACCCTGTAGATCCGAATTTGTG-3′
miR-345	5′-GCTGACTCCTAGTCCAGGGCTC-3′
miR-409	5′-AGGTTACCCGAGCAACTTTGCAT-3′
miR-206	5′-TGGAATGTAAGGAAGTGTGTGG-3′

**Table 3:** Primers used for mRNA Expression Analysis.

Genes	Primer sequences
PTEN	5′-TGGATTCGACTTAGACTTGACCT-3′ (forward)
	5′-TTTGGCGGTGTCATAATGTCTT-3′ (reverse)
PDCD4	5′-TAAGTGACTCTCTCTTTTCCGGT-3′ (forward)
	5′-TTTTTCCTTAGTCGCCTTTTTGC-3′ (reverse)
BCL2	5′-GAACTGGGGGAGGATTGTGG-3′ (forward)
	5′-CCGGTTCAGGTACTCAGTCA-3′ (reverse)
CCND1	5′-GCTGCGAAGTGGAAACCATC-3′ (forward)
	5′-CCTCCTTCTGCACACATTTGAA-3′ (reverse)
CCNE1	5′-GAGCCAGCCTTGGGACAATAA-3′ (forward)
	5′-GCACGTTGAGTTTGGGTAAACC-3′ (reverse)
CDK2	5′-CCAGGAGTTACTTCTATGCCTGA-3′ (forward)
	5′-TTCATCCAGGGGAGGTACAAC-3′ (reverse)
CDK4	5′-ATGGCTACCTCTCGATATGAGC-3′ (forward)
	5′-TAGGCACCGACACCAATTTCA-3′ (reverse)
CDK6	5′-GCTGACCAGCAGTACGAATG-3′ (forward)
	5′-GCACACATCAAACAACCTGACC-3′ (reverse)
β-actin	5′-CATGTACGTTGCTATCCAGGC-3′ (forward)
	5′-CTCCTTAATGTCACGCACGAT-3′ (reverse)

### Immunoblot Analysis

Total cell lysates (25 µg) were separated by SDS-PAGE, transferred to polyvinylidene difluoride membranes (Millipore) and immunoblotted with the indicated antibodies, followed by IRDye800CW goat anti-mouse IgG or IRDye680 goat anti-rabbit IgG (LI-COR Biosciences). Blots were visualized on an Odyssey Infrared Imaging System (LI-COR Biosciences).

### Apoptosis Assay

The induction of apoptosis was monitored by immunoblotting whole cell extracts for caspase-dependent PARP cleavage as previously described [14], or flow cytometry (Accuri Model 6C) using the Annexin V-FITC apoptosis detection kit (BD Pharmingen), according to the manufacturer’s instructions.

### Immunofluorescence and Confocal Microscopy

DU145 cells grown on 8-well glass chamber slides (Millipore) were treated with EF24 (5 µM) for 24 hr. After washing with PBS, slides were fixed with 4% paraformaldehyde/PBS containing 0.3% Triton-X100 for 30 min at 25°C, blocked with 10% goat serum and 1% BSA/PBS for 1 hr and incubated with the indicated primary antibody overnight at 4°C. After incubation with goat anti-rabbit (or rabbit anti-mouse) Alexa Fluor 488 secondary antibody at 25°C for 90 min, DNA was counterstained with Vectashield mounting media with DAPI (Vectra Laboratories). Images were captured on a Zeiss LSM700 laser scanning confocal microscope.

### EMSA Analysis

Nuclear extracts were prepared as previously described and incubated with a ^32^P-labeled κB probe derived from a NF-κB binding sequence in the immunoglobulin gene promoter or the STAT binding site in the SIE derived from the c-fos gene [15]. To define the presence of specific NF-κB or STAT proteins, nuclear extracts were preincubated with a 1:50 dilution of the indicated antibody at 25°C for 0.5 h and then subjected to EMSA. Gels were quantified by PhosphorImage autoradiography.

### Tumor Formation in Mice

All animal experiments were performed in accordance with a study protocol approved by the Institutional Animal Care and Use Committee of the University of Tennessee Health Science Center. Prostate cancer xenografts were established in five-week-old male NOD.Cg-*Prkdc^scid^ Il2rg^tm1Wjl^*/SzJ (NSG) mice (Jackson Laboratory) by injection directly into the flanks of 1×10^6^ DU145 cells transduced with luciferase lentivirus constructs [13]. For analysis of melanoma metastasis, C57/BL6 mice were injected via tail vein with B16 melanoma cells (1×10^6^) in 0.2 ml culture medium [13]. For bioluminescence imaging, mice were injected intraperitoneally with d-luciferin, imaged on the IVIS *in vivo* imaging system (Caliper Life Sciences), and photonic emissions assessed using Living image® software. At ∼7 days after cell injection, when tumors formed in all mice, mice were given daily intraperitoneal injections of either EF24 (200 µg/kg body weight) in 5% Na_2_HCO_3_ buffer or buffer alone. For survival analysis mice were observed daily after injection and were sacrificed at the first sign of shortness of breath, decreased locomotion or reduced body weight (>20% of total body weight). Tumors were measured weekly with a handheld caliper and by bioluminescence imaging. At the end of treatment the animals were sacrificed, and the tumors were removed, weighed and subjected to analysis by immuofluorescent staining on Zeiss LSM700 laser scanning confocal microscope or gene expression analysis by qPCR.

### miRNA Expression Profiling

Total RNA was prepared from DU145 cells treated with EF24 (5 µM, 24 hr) or tumor tissue from DU145 xenografts of EF24-treated mice, and miRNA expression profiling was conducted on the nCounter Analysis System (NanoString Technologies) using the human V1 miRNA assay kit, containing ∼700 human and human-associated viral miRNAs. In brief, total RNA was mixed with pairs of capture and reporter probes, hybridized on the nCounter Prep Station, and purified complexes were quantified on the nCounter digital analyzer. To account for differences in hybridization and purification, data were normalized to the average counts for all control spikes in each sample and analyzed with nSolver software.

### Statistical Analysis

At least three independent experiments were performed in duplicate, and data are presented as means ± sd. ANOVA and post-hoc least significant difference analysis or Student *t* tests were performed. *p* values <0.05 (*), 0.01 (**) and 0.001 (***) were considered statistically significant.

## Results

### EF24 Promotes Apoptosis and Enhances the Effect of miR-21 Knockdown

**Figure 1:**
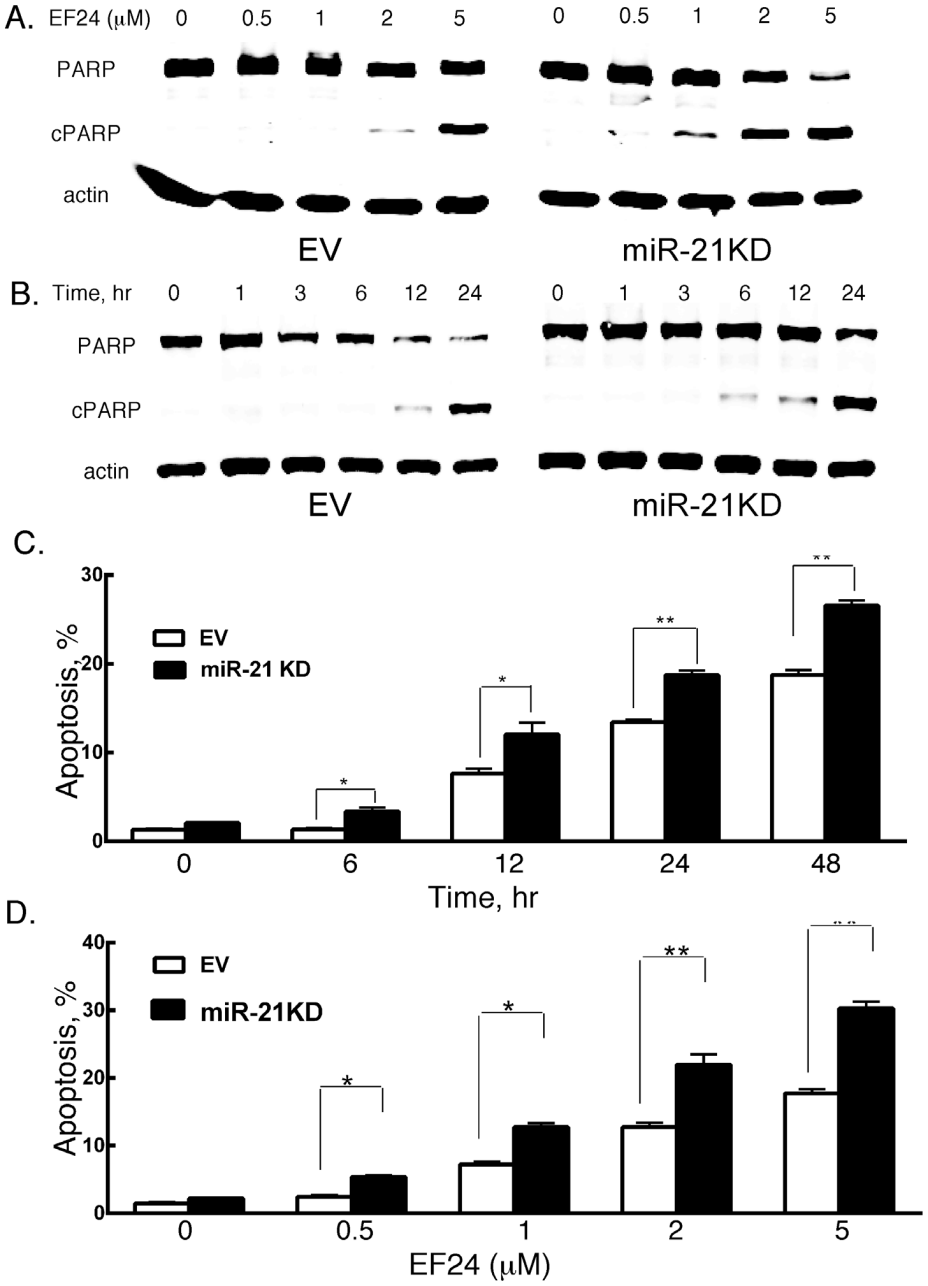
The effects of EF24 and miR-21 KD on apoptosis *in vitro*. EV and miR-21KD DU145 cells were treated with EF24 (A) at varying concentrations for 24 hr or (B) at 5 µM for varying times, and the extent of PARP cleavage (cPARP) as a measure of apoptosis was determined by immunoblotting with anti-PARP. EV and miR-21KD B16 cells were treated with EF24 (C) at 5 µM for varying times or (D) at varying concentrations for 48 hr, and the fraction of annexin V positive cells was determined by flow cytometry.

Previous studies have demonstrated that EF24 induces apoptosis in ovarian, gastrointestinal and breast cancer models [16]–[18]. To determine the ability of EF24 to promote apoptosis in prostate cancer, human DU145 cells were treated with varying EF24 concentrations for 24 hr and PARP cleavage was quantified in cell lysates by immunoblotting. As shown in Figure 1A, PARP cleavage was not observed at EF24 concentrations <2 µM and marked PARP cleavage was found at 5 µM of EF24. Based on these findings, EF24 was used at 5 µM in all subsequent *in vitro* studies of DU145 cells. We previously showed that the miRNA, miR-21, plays an important role in cancer cell survival, and that knockdown (KD) of miR-21 sensitized prostate cancer cells to several chemotherapeutic agents [5]. To determine if miR-21 KD enhanced the sensitivity of DU145 cells to EF24-induced apoptosis, stable pools of DU145 cells with miR-21 levels knocked down by ∼80% were exposed to varying EF24 concentrations. As shown in Figure 1A, miR-21 KD markedly sensitized DU145 to apoptosis with marked PARP cleavage detected at 1 µM of EF24 and PARP cleavage peaked at 2 µM of EF24. We next examined the induction of PARP cleavage at various times after the addition of EF24. As shown in Figure 1B, while PARP cleavage was first detectable at 12 hr in DU145 cells, PARP cleavage was detectable in miR-21KD cells within 6 hr of EF24 addition. To further characterize EF24-promoted apoptosis, DU145 cells were stained with Annexin V at 24 hrs after EF24 addition and subjected to flow cytometric analysis (Figure S1). EF24 treatment markedly augmented the fraction of Annexin V-positive DU145 cells. Moreover, miR-21 KD not only enhanced basal apoptosis in DU145 cells but also sensitized cells to EF24-induced apoptosis. Taken together these results show that EF24 induced apoptosis in DU145 cells and that miR-21 KD enhanced the sensitivity of DU145 to EF24-induced apoptosis.

To further examine the effect of EF24 on apoptosis in another cancer model we employed mouse B16 melanoma cells, a cell line we have previously studied [5], [13], [19]. In brief, B16 cells were treated with 5 µM EF24 and the percentage of apoptotic cells was determined by flow cytometry. As shown in Fig. 1C, apoptosis of B16 cells in response to EF24 treatment was detectable within 12 hr, and continued to 48 hr after treatment. Similar to the findings in DU145 cells (Fig. 1A and B), the appearance of apoptosis was hastened in stable pools of miR-21KD B16 cells, so that apoptosis was detectable within 6 hr in miR-21KD B16 cells (Fig. 1C). Moreover, miR-21 KD also sensitized B16 cells to EF-24 induced apoptosis with increased apoptosis found at 0.5 µM EF24 in miR-21KD cells versus 1 µM in empty vector-transduced B16 cells (Fig. 1D).

### EF24 is a Selective Inhibitor of the NF-κB Pathway, but does not Affect the STAT Pathway

**Figure 2:**
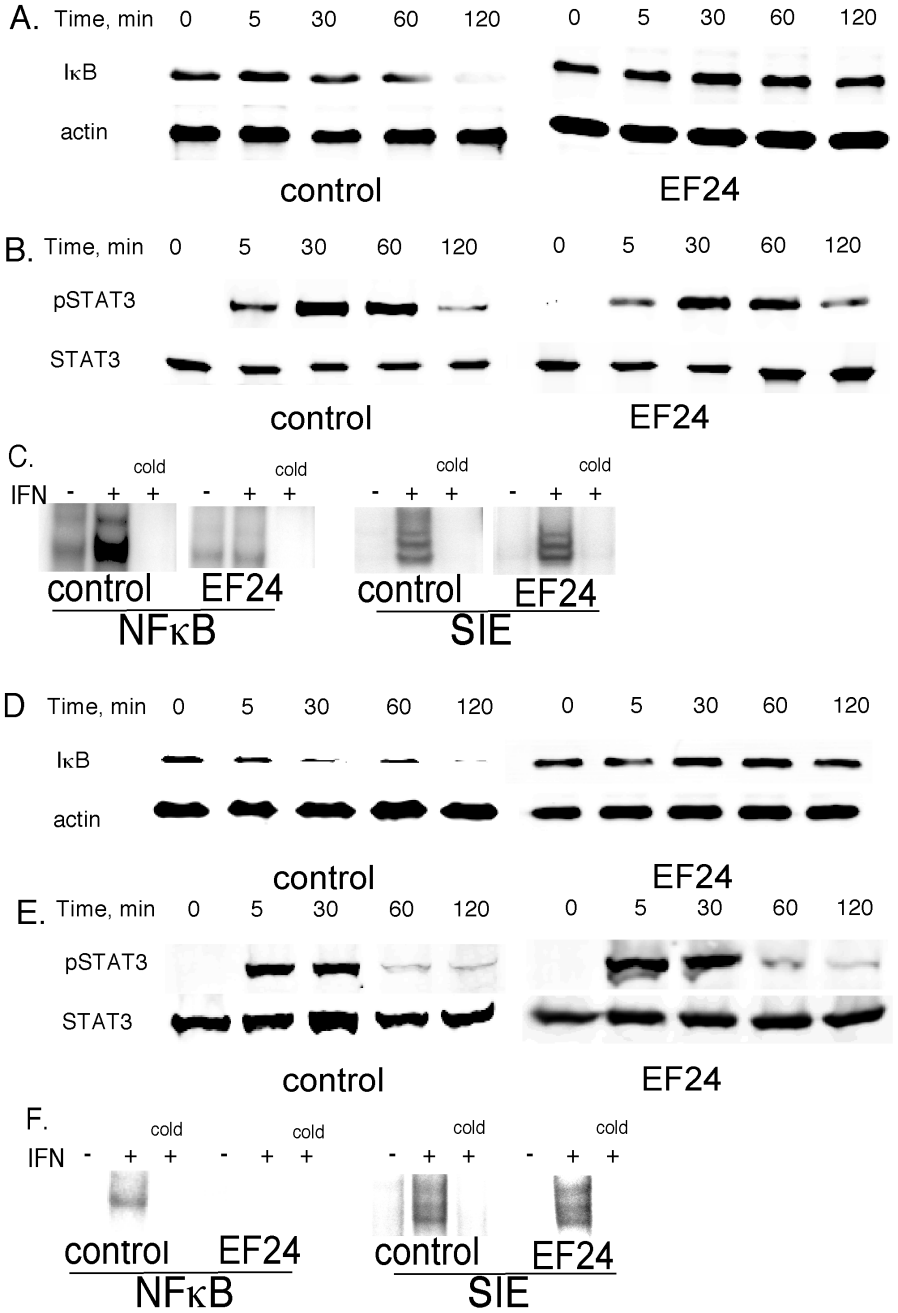
The effects of EF24 on STAT and NF-κB activation *in vitro*. DU145 (A,B) and B16 (D,E) cells were treated with EF24 (5 µM) or vehicle (control) for 24 hr prior to stimulation with IFN (1000 IU/ml) for the indicated times. Whole cell extracts were prepared and immunoblotted for (A,D) IκB and actin, or (B,E) phosph-STAT3 and STAT3. (C,F) Nuclear extracts were prepared from IFN-stimulated cells (1000 IU/ml for 30 min) DU145 (C) or B16 (F) cells pretreated for 24 hr with EF24 (5 µM) or vehicle (control) and subjected to EMSA with oligonucleotide probes for NF-κB or SIE. A 50-fold excess of unlabeled probe (cold) was used to show the specificity of binding. Representative results from at least three experiments are shown.

EF24 has been found to inhibit the NF-κB pathway, but its impact on the STAT pathway has not been examined. Since curcumin has been shown to inhibit both STAT3 and NF-κB activation [20], we examined the effects of EF24 on these signaling pathways. To induce both STAT3 and NF-κB activation, human DU145 prostate cancer cells and murine B16 melanoma cells were stimulated with human and mouse type I IFN, respectively, a potent activator of these signaling pathways [15], [21]. Cellular extracts were prepared from cells pretreated with EF24 (5 µM for 24 hr) prior to IFN addition and subjected to immunoblotting. As shown in Figure 2A and D, and consistent with our previous findings [22], IFN induced a decrease in cellular IκBα levels in DU145 and B16 cells within 30 min of IFN addition, indicative of NF-κB activation. In contrast, EF24 pretreatment completely blocked the IFN-induced decrease in cellular IκBα levels. As shown in Figure 2B and E, although IFN treatment induced a robust activation of STAT3, EF24 had no detectable effect of IFN-induced STAT3 activation.

**Figure 3:**
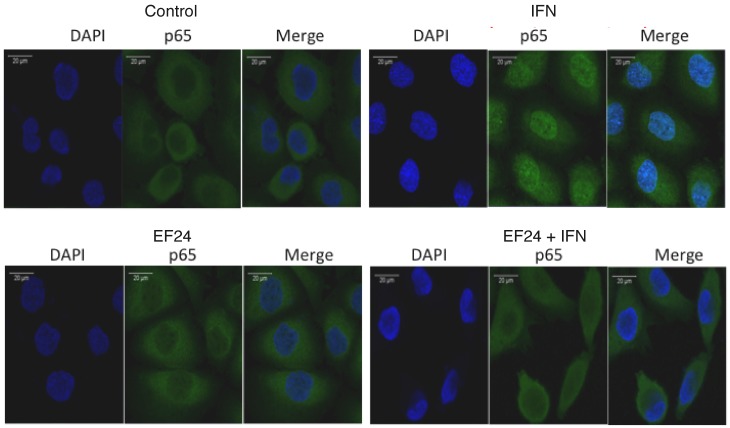
The effects of EF24 on activation of the p65 subunit of NF-κB in DU145 cells *in vitro*. DU145 cells plated on 8-well chamber slides were treated with EF24 (5 µM) or vehicle (control) for 24 hr prior to stimulation with human IFNα (1000 IU/ml) for 30 min. Cells were fixed with 4% paraformaldehyde and methanol, and permeabilized with 1% Triton X100. After blocking with 5% goat serum, slides were stained for p65 and mounted using Vectashield medium with DAPI, and images were captured on a Zeiss LSM700 laser scanning confocal microscope.

To determine directly whether EF24 blocks NF-κB activation and further characterize the effect of EF24 on STAT activation we examined activation of these signaling pathways by DNA binding assays. In brief, DU145 and B16 cells were pretreated with EF24 prior to IFN addition, nuclear extracts were prepared, incubated with the appropriate oligonucleotide probes and subjected to EMSA. As shown in the left hand panels of Figure 2C and F, IFN induced a robust activation of NF-κB activity in both DU145 and B16 cells, which is consistent with our previous findings in other cells [22]. The NF-κB complex was supershifted by both anti-p50 and anti-p65 antibodies, demonstrating that the IFN-induced complex is comprised of p50:p65 heterodimers (Figure S2). A 50-fold excess of unlabeled oligonucleotide probe abrogated the binding of NF-κB complex, showing that NF-κB binding was indeed specific. Most importantly, EF24 pretreatment of either cell line completely blocked the IFN-induced activation of the NF-κB complex. These results are consistent with the ability of EF24 to block the IFN-induced decrease in cellular IκBα levels (Fig. 2A and D). In contrast to the inhibitory effect of EF24 on the NF-κB pathway, EF24 had no effect of IFN-induced STAT DNA binding activity (right hand panels of Figure 2C and F). As shown in Figure S2, IFN induced both STAT1- and STAT3-containing DNA binding complexes that were supershifted by anti-STAT1 and STAT3 antibodies. However, the induction of STAT-dependent DNA binding complexes by IFN was unaffected by EF24 pretreatment. The specificity of STAT binding was evidenced by competition with excess oligonucleotide probe. Moreover, EF24 abrogated the IFN-induced nuclear translocation of the p65 subunit of NF-κB in DU145 cells as evidenced by immunoflourescent staining and confocal microscopy (Figure 3). Taken together, these results demonstrate that EF24 selectively blocks the NF-κB pathway in cancer cells, but does not affect STAT1 or STAT3 activation.

### EF24 Inhibits miR-21 Expression and Increases the Expression of miR-21 Target Genes

**Figure 4:**
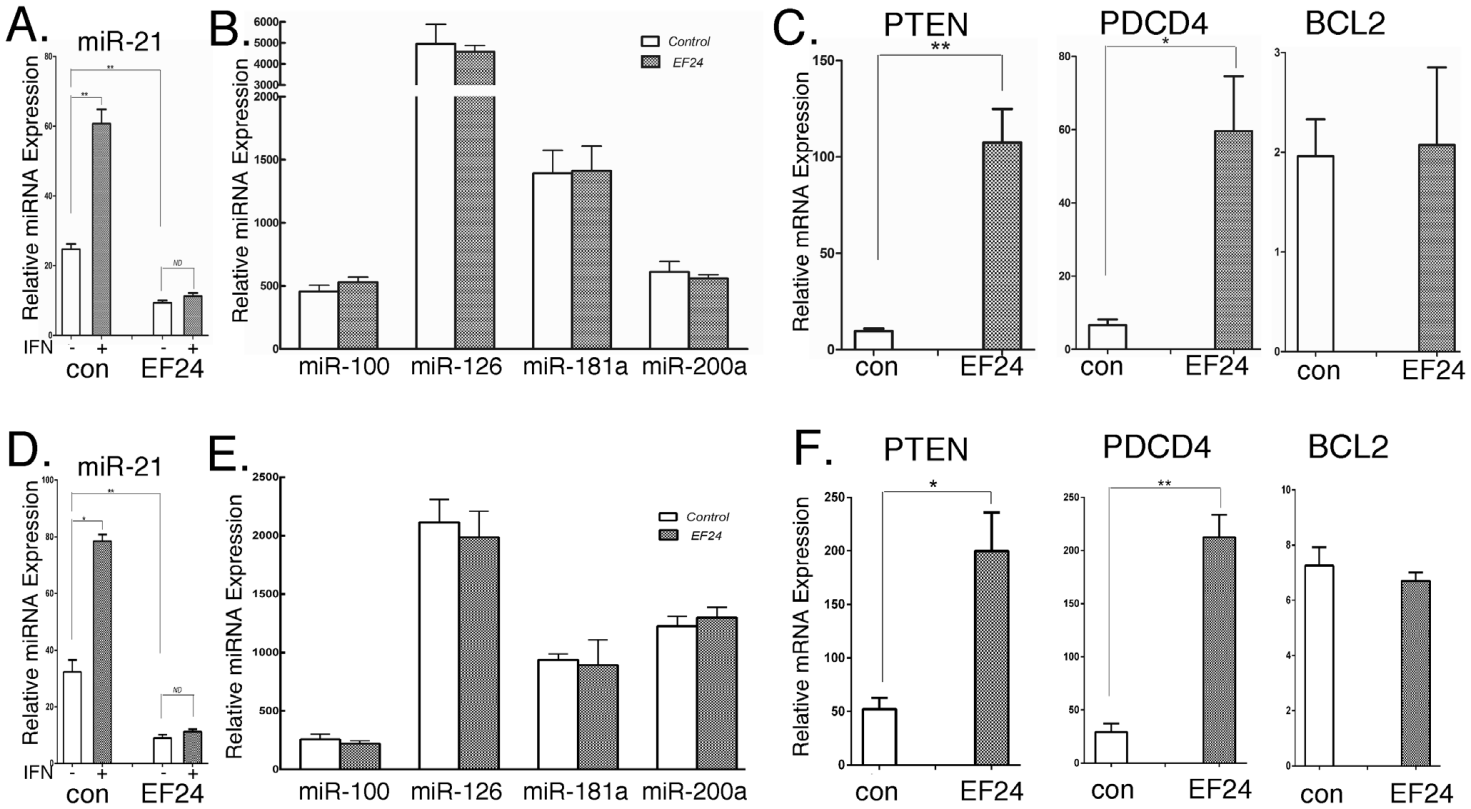
The effects of EF24 on miRNA expression and miR-21 target gene expression in DU145 and B16 cells *in vitro*. DU145 (A-C) and B16 (D-F) cells were treated with EF24 (5 µM for 24 hr) and total RNA assayed for (A,B,D and E) miRNA or (C,F) mRNA expression by qPCR (n = 3). The effect of EF24 treatment on IFN-induced (1000 IU/ml for 6 hr) miR-21 expression was also assessed. (A,D).

**Figure 5:**
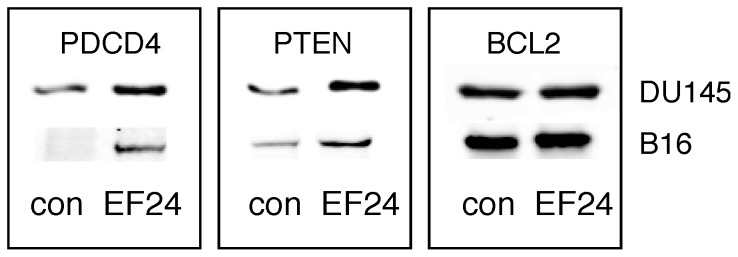
The effects of EF24 miR-21 target protein expression in DU145 and B16 cells *in vitro*. DU145 and B16 cells were treated with 5 µM EF24. At 24 hr after EF24 treatment cells were lysed, and the expression of miR-21 target proteins was determined by immunoblotting.

Based on the findings presented so far and having previously shown that miR-21 gene expression is STAT3 and NF-κB dependent [5], we examined the effect of EF24 on miR-21 gene expression. In brief, total cellular RNA was prepared from DU145 and B16 cells treated with EF24 (5 µM for 24 hr), and miRNA levels were determined by quantitative real time PCR (qPCR). As shown in Figure 4A and D EF24 treatment of DU145 and B16 cells, respectively, reduced miR-21 levels by >70%, while it had no effect on the expression levels of miR-100, -126, -181a and -200a (Fig. 4B and E). Moreover, EF24 treatment nearly completely abrogated the IFN-induced increase in miR-21 (Fig. 4A and D). PTEN, PDCD4 and BCL2 have been previously characterized as miR-21 target genes [23]–[26]. Therefore, we examined the effect of EF24 on the expression of these miR-21 targets by qPCR in DU145 and B16 cells and immunoblotting. EF24 treatment resulted in a marked increase in PTEN and PDCD4 gene expression (Figure 4C and F) in DU145 and B16 cells, respectively,, but had no effect on BCL2 expression. Moreover, EF24 treatment also increased PDCD4 and PTEN expression at the protein level, but not that of BCL2 (Fig. 5). These findings are consistent with our previous studies showing that miR-21 target genes are highly cell type specific [5], [13]. Taken together, these results show that miR-21 and miR-21-target genes are affected by EF24 treatment.

### EF24 has Potent Anticancer Activity against Prostate Cancer *in vivo*

**Figure 6:**
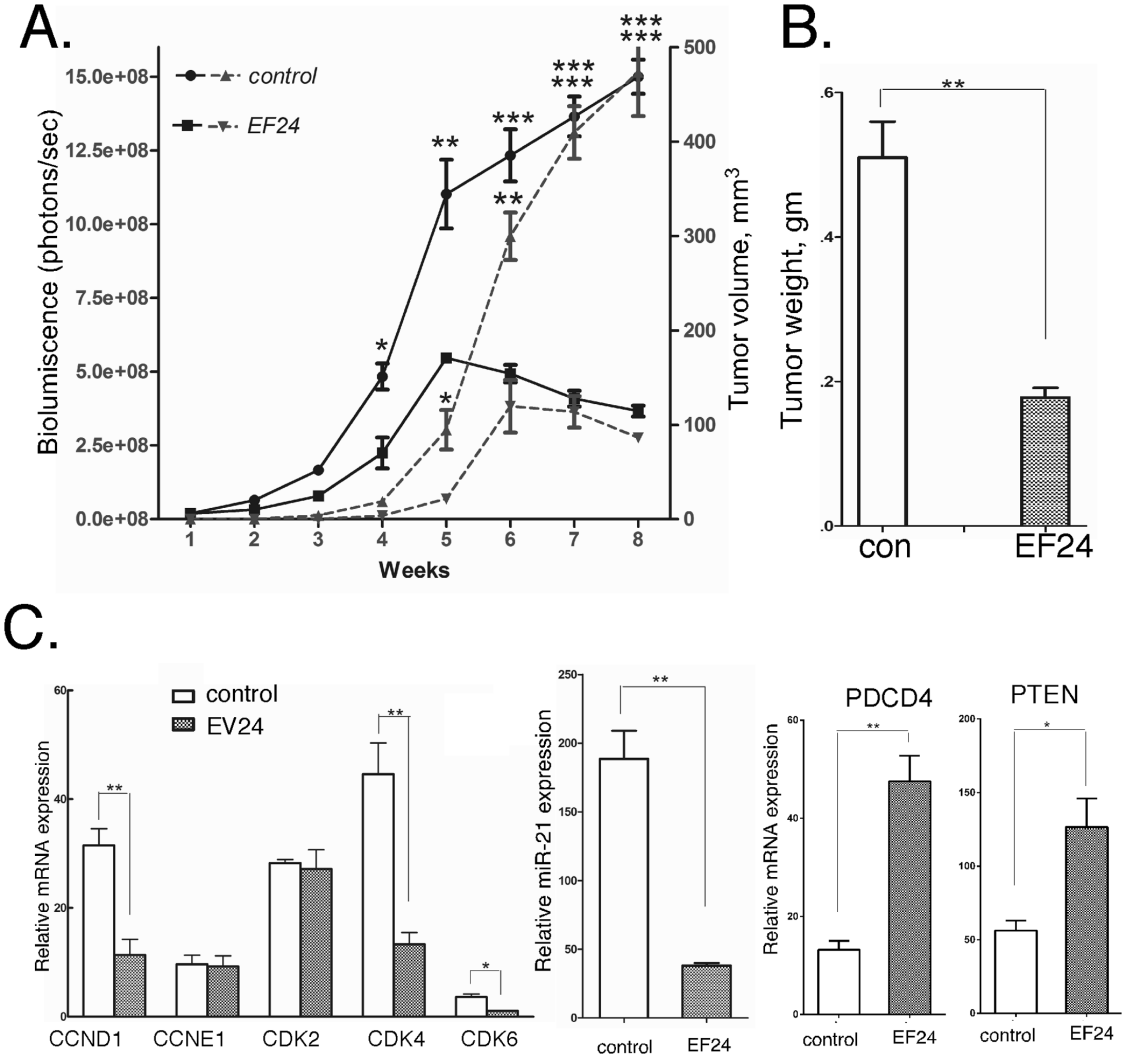
EF24 inhibits prostate cancer tumor xenograft growth *in vivo*. (A,B) NSG mice were injected subcutaneously with 10^6^ DU145 cells, and after tumor engraftment mice were injected daily with EF24 (200 µg/kg body weight). (A) Tumor growth was determined by bioluminescent imaging of mice injected with luciferin (solid line) or by caliper measurements (dashed line) (n = 8). (B) At necropsy tumors were weighed, or (C) RNA was extracted from tumor tissue and subjected to gene expression analysis by qPCR as indicated (n = 3).

**Figure 7:**
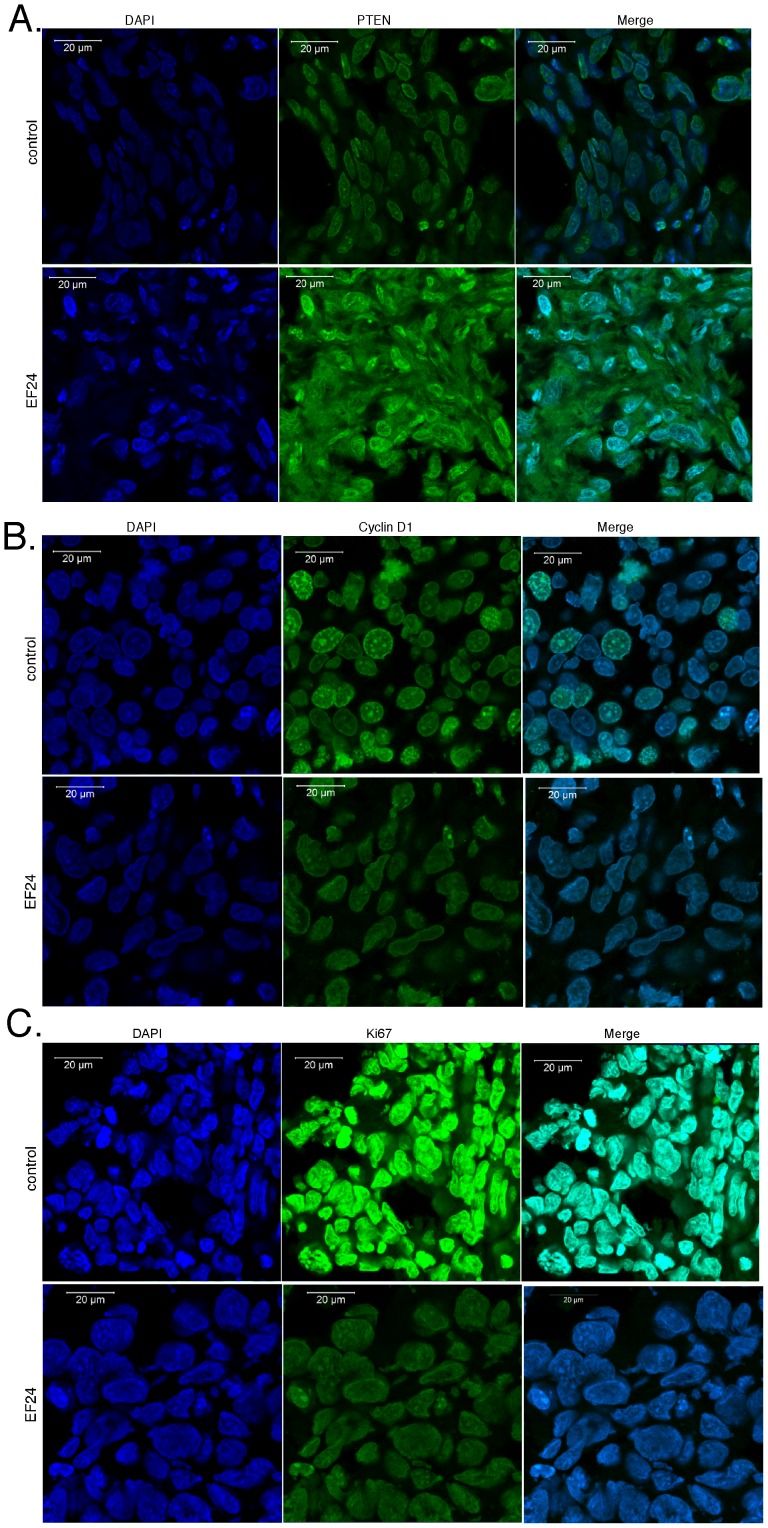
The effects of EF24 on the expression of key target genes in prostate cancer tumor xenografts *in vivo*. At the end of treatment (∼8 weeks) the control and EF24-treated mice (200 µg/kg body weight) injected with DU145 cells were sacrificed, and the tumors were removed, sectioned with a cryostat and immunostained for (A) PTEN, (B) Cyclin D1, or (C) Ki67. Sections were imaged on a laser scanning confocal microscope.

We then examined the effect of EF24 against prostate cancer *in vivo*. In brief, NSG mice were injected subcutaneously with DU145 cells engineered to express luciferase for noninvasive bioluminescence live animal imaging (1×10^6^ cells per injection site). Once a palpable mass was detectable (∼1 week), mice were then subjected to daily intraperitoneal injections of EF24. The dose of EF24 (200 µg/kg body weight) was selected based on a previous study on colon cancer xenografts [11]. Tumor volume in control (vehicle) and EF24-treated mice was determined at weekly intervals by caliper measurement and bioluminescent imaging. As shown in Figure 6A, DU145 tumors formed rapidly (within one-week) and grew continuously during the 8-week preclinical trial with bioluminescence imaging a more sensitive measure of tumor growth than caliper measurement. Most importantly, EF24 treatment markedly slowed tumor growth and resulted in tumor regression by 4 weeks of treatment. The ∼65% reduction in tumor mass in the EF24-treated mice was further evidence of the antitumor efficacy of EF24 (Figure 6B). To investigate whether the effects of EF24 in an animal model are consistent with its anticancer activity, the expression of several cell cycle regulated genes as well as miR-21 and miR-21-target genes was quantified in RNA extracts of DU145-derived tumor tissue. As shown in Figure 6C, EF24 treatment resulted in the decreased gene expression of CCND1, CDK4 and CDK6, but not of CCNE1 or CDK2. Consistent with the effects of EF24 on cell cycle related proteins, EF24 treatment of DU145 cells *in vitro* results in a G1 arrest as assayed by flow cytometry of propidium iodide-stained cells (Figure S3). Not surprisingly, miR-21 gene expression was markedly reduced (∼70%), while the expression of PTEN and PDCD4 (miR-21-target genes) was enhanced in tumor tissue from EF24-treated mice, which is similar to the effect of EF24 on miR-21 gene expression in DU145 cells *in vitro* (Figure 6C). In addition, the effects of EF24 on the expression of several growth-related genes as well as miR-21 target gene expression by immunostaining of tumor tissue collected from EF24 treated mice injected with DU145 cells. As shown in Figure 7A, the expression of the miR-21 target gene PTEN was markedly increased in tumor tissue from EF24-treated mice when compared to controls. In contrast, the expression of two markers of proliferating cells, cyclin D1 (Fig. 7B) and Ki67 (Fig. 7C), were markedly reduced in tumors from EF24 mice.

### EF24 has Potent Anticancer Activity against Melanoma *in vivo*

**Figure 8:**
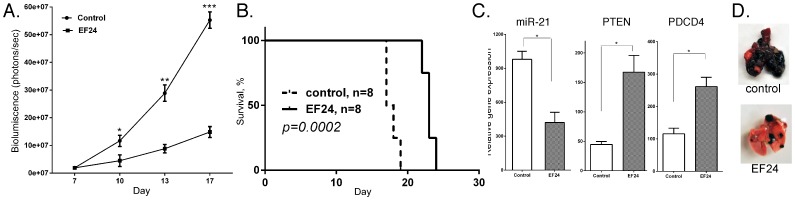
EF24 treatment inhibits the tumorigenic potential of B16 melanoma cells *in vivo*. Mice were injected with 10^6^ B16 cells into the tail vein and after 1 week were injected daily with EF24 (200 µg/kg body weight). (A) Bioluminescent imaging of mice injected with luciferin performed at the indicated days, and (B) Kaplan-Meier analysis of survival data (n = 8). (C) RNA was extracted from tumor tissue at necropsy and subjected to gene expression analysis by qPCR as indicated (n = 3). (C) Lungs of control and EF24 treated mice at necropsy (17 days post-tail vein injection).

B16 melanoma cells form lung metastases when injected into the tail-vein of syngeneic C57BL6 mice, and serve as a model for melanoma metastasis [27]–[29]. Therefore, we sought to further characterize the anticancer activity of EF24 by examining its effect on B16 cells *in vivo*. In brief, B16 cells transduced with Luc-2 for bioluminescent imaging were introduced into the bloodstream of syngeneic C57BL6 mice by tail-vein injection. EF24 treatment was initiated at one week post-injection, when bioluminescent imaging showed that a strong luciferase signal was detectable in the lungs of all mice as previously shown [13], and EF24 treatment was continued daily. A dramatic increase (∼550-fold) in bioluminescent signal intensity was detected at 17 days post-injection in the lungs, while only a relatively weak signal was detected in the lungs of EF24-treated mice (Fig. 8A). As further evidence of the anticancer activity of EF24 on B16 cells, EF24 treatment markedly prolonged the survival of mice injected with B16 cells (Fig. 8B). Mice injected with B16 cells had a mean survival of ∼17.5 days, while EF-24–treated mice had a mean survival of ∼23.3 days. Upon necropsy, while miR-21 expression was markedly diminished in lung tumor tissue from EF24-treated mice, the expression of miR-21 target genes (PTEN and PDCD4) was enhanced (Fig. 8C). In addition, EF24 treatment reduced the number and size of metastatic lesions in the lungs of mice (Fig. 8D).

### EF24 Selectively Regulates miRNA Expression

**Figure 9:**
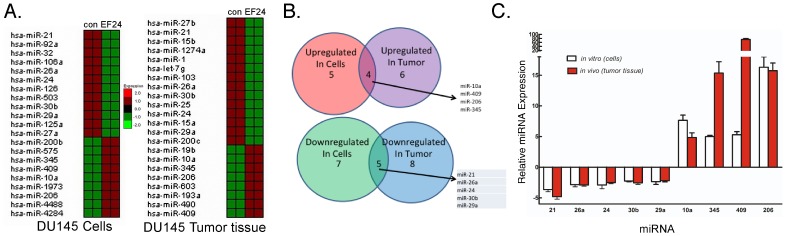
The effects of EF24 on miRNA expression *in vitro* and *in vivo*. (A) Total RNA was prepared from DU145 cells treated with vehicle and EF24 (5 µM, 24 hr), or tumor tissue from DU145 xenografts of control and EF24-treated mice (200 µg/kg body weight) at autopsy, and miRNA expression profiling was conducted on the nCounter Analysis System using the human V1 miRNA assay kit. (B) Venn diagram analysis of the genes upregulated or downregulated by EF24 treatment in cells and tumor tissue. (C) PCR validation of the genes upregulated or downregulated by EF24 treatment in both cells and tumor tissue (n = 3), and expressed as relative gene expression (EF24-treated/control).

Although EF24 treatment decreased miR-21 expression, we sought to determine whether other miRNAs might also be regulated by EF24. Using the nCounter platform to assay for the expression of human miRNAs, we performed miRNA profiling of RNA prepared from EF24-treated DU145 cells and tumor tissue derived from EF24-treated mice injected with DU145 cells. The miRNA signature from EF24-treated mice was compared that of vehicle-treated mice, and the miRNA signature from EF24-treated cells was compared to the profile from untreated control cells (Fig. 9A). As shown in Figure 9B the expression of 21 miRNAs were affected by EF24 treatment of DU145 cells *in vitro*, and the expression of 23 miRNAs were affected by EF24 *in vivo* in DU145-derived tumor tissue. Only four miRNAs (miR-10a, miR-409, miR-206 and miR-345) were upregulated both *in vitro* and *in vivo*, which reportedly act as tumor suppressors or inhibitors of cell cycle progression. In contrast, only 5 miRNAs (miR-21, miR-26a, miR-24, miR-30b and miR-29a) were found to be downregulated both *in vitro* and *in vivo* by EF24 treatment. These miRNAs have been found to act as oncogenic miRNAs (also called oncomirs) as well as enhancers of cell proliferation. Among this group of downregulated miRNAs was miR-21, which validates this approach as we have already shown that miR-21 expression is inhibited by EF24 treatment. To independently verify that these miRNAs were regulated by EF24, we performed qPCR for these miRNAs in RNA extracts from DU145-derived tumor tissue or DU145 cells grown *in vitro*. As shown in Figure 9C, there was excellent concordance in the data from the miRNA profiling and qPCR, the expression of miR-21, miR-26a, miR-24, miR-30b and miR-29a was down-regulated by EF24 treatment both *in vitro* and *in vivo*, while the expression of miR-345, miR-409, miR-10a and miR-206 was upregulated by EF24 treatment.

## Discussion

Epidemiological studies suggest that besides race and age, diet is also an important risk factor for various forms of cancer [30]. While a high fat diet is implicated in cancer, polyphenolic compounds present in common plant-derived foods have long been recognized for their anticancer properties [31]. Most notably curcumin, the yellow pigment in the spice turmeric, has anticancer activity in various pre-clinical cancer models *in vitro* and *in vivo*. However, since curcumin has diverse biological effects and targets multiple signal transduction pathways including NF-κB and STAT3, poor bioavailability, and relatively low anti-cancer potency, several more selective and potent analogs of curcumin have been developed [11], [32]. EF24 is a curcumin analog with potent *in vitro* and *in vivo* anticancer activity in hepatocellular carcinoma, head and neck squamous carcinoma, and breast cancer [16]–[18]. In the present study we show that EF24 also has potent anticancer activity in both prostate cancer and melanoma models. In contrast to curcumin, we found that EF24 inhibits the NF-κB pathway but has no effect on STAT activation. We found that EF24 inhibited not only basal NF-κB activation but also activation induced by the cytokine IFN. While IFN stimulated STAT1, STAT3 and NF-κB activation, EF24 only inhibited IFN-induced NF-κB activation as shown by immunoblotting for cellular IκB levels and DNA binding assays. This is of particular interest because of the crosstalk of these pathways in various cancers [33], as well as their activation in clinical prostate cancer and melanoma samples [9], [10]. Our results indicate that EF24 selectively targets the NF-κB pathway.

In addition, we found that EF24 promotes the apoptosis of prostate cancer and melanoma cells, which in part reflects the EF24 induced down-regulation of the oncogenic miRNA miR-21. EF24 has been previously shown to promote the apoptosis of various other cancer cell lines [11]. Moreover, knockdown of miR-21 not only hastens the time course of apoptosis induced by EF24, but also results in the induction of apoptosis at lower EF24 concentrations. Since we previously found that DU145 and B16 cells have relatively high levels of miR-21 [5], our present results indicate that prolonged EF24 treatment results in reduced miR-21 expression, which sensitizes cancer cells to EF24-induced apoptosis. miR-21 is considered an oncomir because it is expressed at higher levels in cancer cell lines and tumor tissue, and plays an important role in cancer cell survival, progression and metastasis [4]. EF24 treatment inhibits miR-21 expression, but does not affect the expression of several other miRNAs, including miR-100, -126, -181a and -200a. Moreover, EF24 inhibits basal as well as IFN-induced miR-21 expression. We previously established that IFN induced the binding of the p65 NF-κB subunit and STAT3 to the miR-21 promoter to regulate miR-21 expression [5]. We show that EF24 treatment not only blocks the induction of p65-containing NF-κB complexes by IFN but also inhibits IFN-induced miR-21 expression, which is consistent with our previous findings on the critical role of p65 in miR-21 expression [5]. As additional evidence that miR-21 is an important target of EF24, EF24 treatment of DU145 cells also results in an increased expression at the mRNA and protein level of known miR-21 target genes, PTEN and PDCD4 [5], [13], [23]–[26].

We also found that EF24 treatment rapidly suppressed the growth of prostate cancer xenografts in mice, and long-term EF24 treatment resulted in tumor regression. We also found that the expression of the miR-21 target protein PTEN was enhanced in tumor tissue isolated from EF24-treated mice, while not surprisingly miR-21 expression was reduced. The antitumor activity of EF24 also appears to reflect a direct effect of EF24 on prostate cancer cell proliferation as the expression of both cyclin D1 and Ki67 was markedly reduced in tumor xenografts from EF24-treated mice.

Moreover, we found that EF24 alters the metastatic behavior of mouse B16 melanoma cells. Upon tail-vein injection vector B16 cells selectively home to the lung, forming rather large tumors [13], [27]–[29]. In contrast, after EF24 treatment B16 cells home to the lung but only form a few micrometastases, and there was a significant increase in the mean survival of EF24-treated mice. These effects of EF24 in the B16 melanoma model were similar to what we previously observed when miR-21KD melanoma cells were injected into syngeneic mice [13]. This point is particularly relevant since we observe a marked decrease in miR-21 expression in B16 lung tumors from EF24 treated mice as well as an enhanced expression of miR-21 target genes, including PTEN and PDCD4. Taken together, these data demonstrate that EF24 treatment results in a diminished metastatic potential of B16 cells. Metastasis is a major cause of cancer-related death, especially in melanoma. Thus, our finding that EF24 inhibits the metastatic properties of melanoma has important clinical implications.

Although miR-21 appears to be an important miRNA target of EF24, we also found that the expression of other miRNAs could be regulated by EF24 treatment. Interestingly, only 5 miRNAs were downregulated by EF24 both by EF24 treatment of DU124 cells and in mice treated with EF24 with DU145 xenografts. This subset included a number of tumor-promoting miRNAs. Among the EF24-downpregulated miRNAs, which non-surprisingly included miR-21, miR-26a can transform cells and promotes glioma proliferation *in vitro* and *in vivo* [34]. miR-24 is a putative oncomir and is overexpressed in breast and cervical carcinoma [35]. miR-30b appears to play an important oncogenic role in the development of medulloblastoma [36]. The expression of miR-29a is elevated in some cancers where it appears to function as an oncogene [37], while in some cancers it may function as a tumor suppressor [38].

There was also a subset of 4 miRNAs that were upregulated under both *in vitro* and *in vivo* conditions, which included a number of miRNAs that may have tumor suppressing or growth inhibiting activities. Among these upregulated miRNAs were miR-10a, which is a candidate tumor suppressor and suppresses apoptosis in leukemia [39], miR-409 that suppresses tumor cell invasion and metastasis in gastric cancer [40], and miR-206 and miR-345, which are frequently downregulated in various types of cancers and are believed to act as tumor suppressors [41], [42]. The effect of EF24 on the expression of these miRNAs was validated by qPCR. Future experimentation is required to determine whether these miRNAs indeed have tumor-promoting or suppressive activity on prostate cancer *in vitro* and *in vivo*. Nonetheless, taken together our results suggest that EF24 has potent anticancer activity in melanoma and prostate cancer, which may be mediated in part by targeting a miR-21 anti-apoptotic pathway.

## Supporting Information

Figure S1 **The effects of EF24 and miR-21 KD on apoptosis of DU145 cells** ***in vitro*** **.** EV and miR-21KD DU145 cells were treated with EF24 at 5 µM for 24 hr and apoptosis was determined by flow cytometry of Annexin V-stained cells. Results from at least three representative experiments are shown.
(DOCX)

Figure S2 **IFN-induced NF-κB and STAT activation in DU145 cells.** Nuclear extracts were prepared from IFN-stimulated cells (1000 IU/ml for 30 min) and subjected to EMSA with oligonucleotide probes for NF-κB or SIE. Supershift assays were performed with anti-p50, p65, STAT1 and STAT3 as indicated. Representative results from at least three experiments are shown.
(DOC)

Figure S3 **The effects of EF24 on cell cycle distribution in DU145 cells** ***in vitro*** **. (**A) Representative cell cycle histograms obtained through propidium iodide staining of DU145 cells that were treated with EF24 (5 µM) or vehicle (control) for 24 hr. Cell cycle analysis was performed by flow cytometry. (B) Average percentage of the cells in G1, S, and G2/M phases of the cell cycle. Data represent the average of three independent experiments.
(DOC)
