# Supplementary material for: The Curcumin Analog EF24 Targets NF-κB and miRNA-21, and Has Potent Anticancer Activity In Vitro and In Vivo
Source: PLoS One. 2013 Aug 7;8(8):e71130. doi: 10.1371/journal.pone.0071130 (PMC3737134; doi:10.1371/journal.pone.0071130)

**Figure S1. The effects of EF24 and miR-21 KD on apoptosis of DU145 cells *in vitro*.** EV and miR-21KD DU145 cells were treated with EF24 at 5 μM for 24 hr and apoptosis was determined by flow cytometry of Annexin V-stained cells. Results from at least three representative experiments are shown.


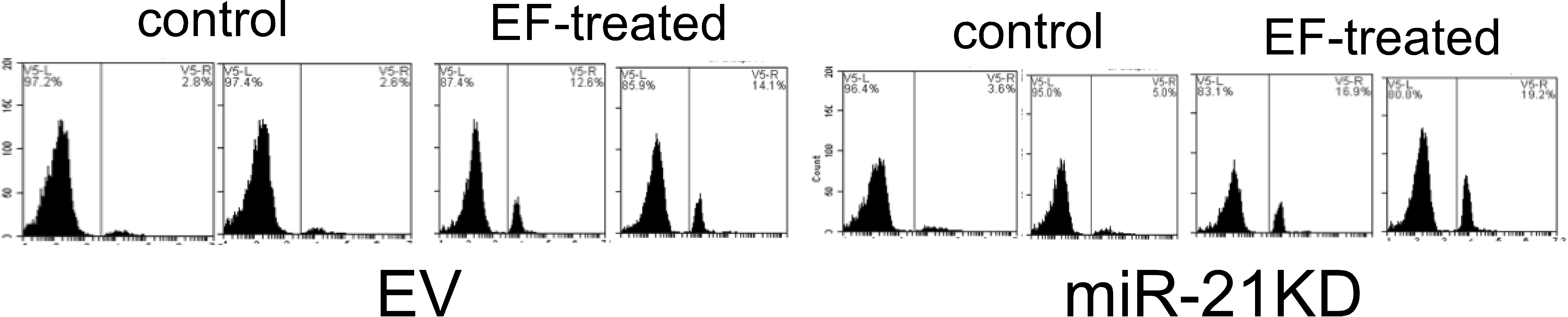

Supplement: Figure S1 — The effects of EF24 and miR-21 KD on apoptosis of DU145 cells in vitro . EV and miR-21KD DU145 cells were treated with EF24 at 5 µM for 24 hr and apoptosis was determined by flow cytometry of Annexin V-stained cells. Results from at least three representative experiments are shown. (DOCX) [file pone.0071130.s001.docx]
