# Supplementary material for: The Curcumin Analog EF24 Targets NF-κB and miRNA-21, and Has Potent Anticancer Activity In Vitro and In Vivo
Source: PLoS One. 2013 Aug 7;8(8):e71130. doi: 10.1371/journal.pone.0071130 (PMC3737134; doi:10.1371/journal.pone.0071130)

**Figure S2. IFN-induced NF-κB and STAT activation in DU145 cells.** Nuclear extracts were prepared from IFN-stimulated cells (1000 IU/ml for 30 min) and subjected to EMSA with oligonucleotide probes for NF-κB or SIE. Supershift assays were performed with anti-p50, p65, STAT1 and STAT3 as indicated. Representative results from at least three experiments are shown.


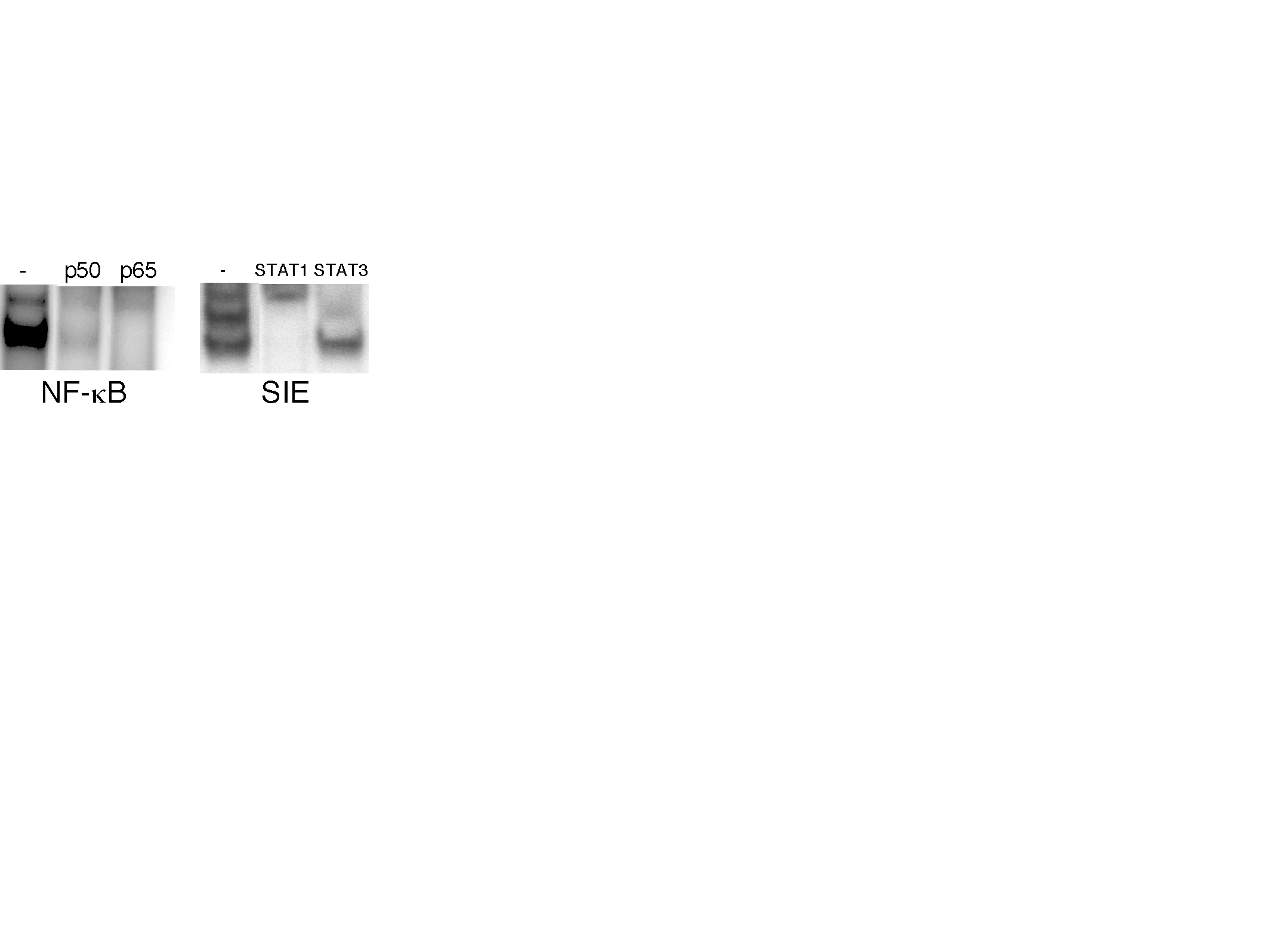

Supplement: Figure S2 — IFN-induced NF-κB and STAT activation in DU145 cells. Nuclear extracts were prepared from IFN-stimulated cells (1000 IU/ml for 30 min) and subjected to EMSA with oligonucleotide probes for NF-κB or SIE. Supershift assays were performed with anti-p50, p65, STAT1 and STAT3 as indicated. Representative results from at least three experiments are shown. (DOC) [file pone.0071130.s002.doc]
