# Supplementary material for: The Curcumin Analog EF24 Targets NF-κB and miRNA-21, and Has Potent Anticancer Activity In Vitro and In Vivo
Source: PLoS One. 2013 Aug 7;8(8):e71130. doi: 10.1371/journal.pone.0071130 (PMC3737134; doi:10.1371/journal.pone.0071130)

**Figure S3. The effects of EF24 on cell cycle distribution in DU145 cells *in vitro*. (**A) Representative cell cycle histograms obtained through propidium iodide staining of DU145 cells that were treated with EF24 (5 μM) or vehicle (control) for 24 hr. Cell cycle analysis was performed by flow cytometry. (B) Average percentage of the cells in G1, S, and G2/M phases of the cell cycle. Data represent the average of three independent experiments.


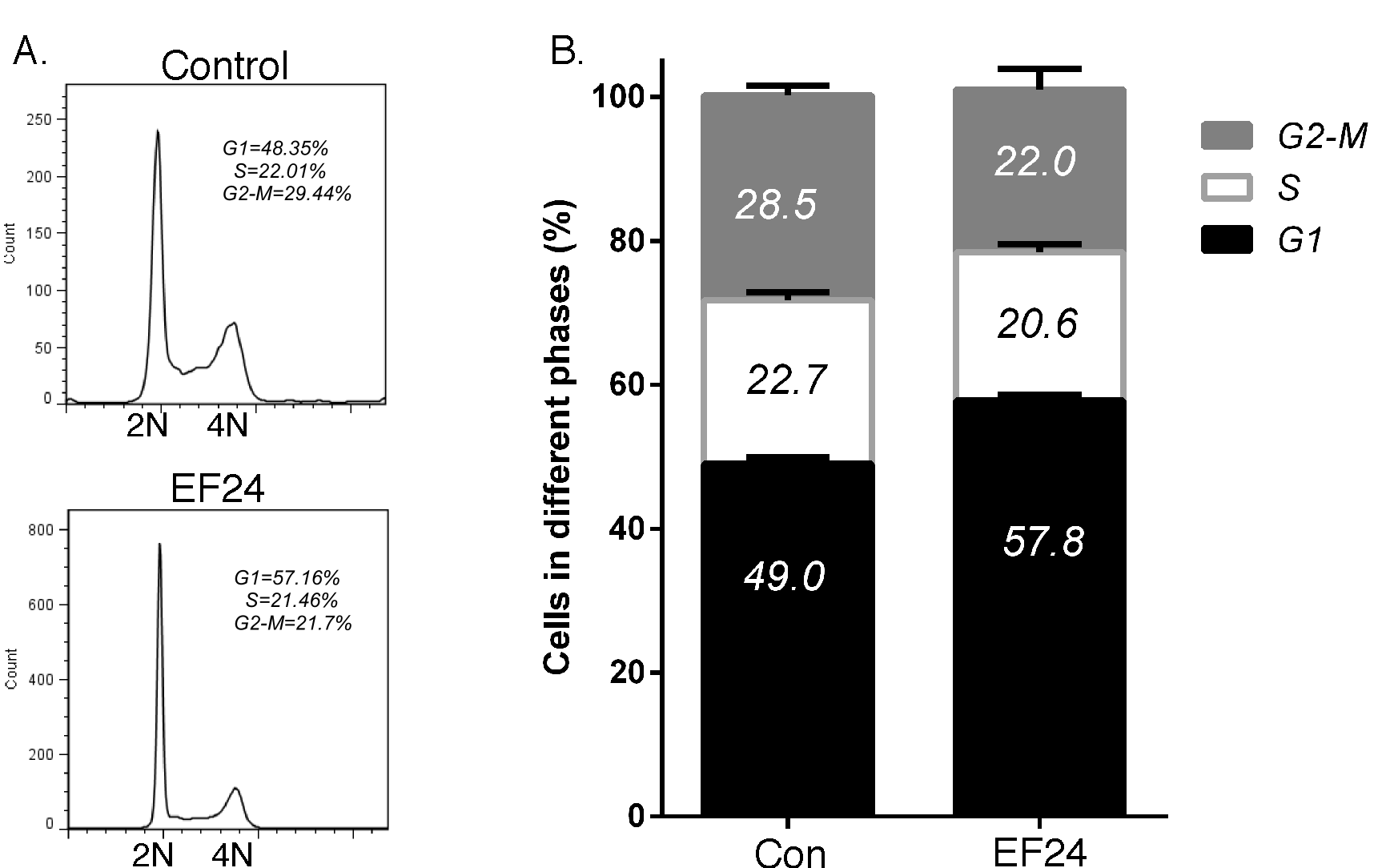

Supplement: Figure S3 — The effects of EF24 on cell cycle distribution in DU145 cells in vitro . (A) Representative cell cycle histograms obtained through propidium iodide staining of DU145 cells that were treated with EF24 (5 µM) or vehicle (control) for 24 hr. Cell cycle analysis was performed by flow cytometry. (B) Average percentage of the cells in G1, S, and G2/M phases of the cell cycle. Data represent the average of three independent experiments. (DOC) [file pone.0071130.s003.doc]
